# Periareolar Extra-Glandular Breast Augmentation

**Published:** 2013-06

**Authors:** Muhammad Humayun Mohmand, Muhammad Ahmad

**Affiliations:** 1 Cosmetic Plastic Surgeon, La Chirurgie, Islamabad Cosmetic Surgery Centre, Islamabad, Pakistan;; 2 Plastic, Reconstructive Surgeon, La Chirurgie, Islamabad Cosmetic Surgery Centre, Islamabad, Pakistan

**Keywords:** Periareolar, Extra-glandular, Breast augmentation

## Abstract

**BACKGROUND:**

Breast augmentation is the most frequent procedure performed according to the 2009 Quick Facts report of the American Society of Plastic Surgeons. This study presents the periareolar extra-glandular breast augmentation.

**METHODS:**

From 2004 to 2010 among 32 female patients, peri-areolar incision was performed for breast augmentation. Dissection was performed in subcutaneous plane towards the inferior pole to reach the inframammary fold and was continued in the upwards direction in the subglandular plane to create a pocket. Once the implant of desired size was in place, three sutures fixed the inframammary fold. The skin incision was closed using 4-0 non-absorbable suture.

**RESULTS:**

The mean age of patients was 30.7 years and the average incision length was 5.8 cm. 59.4% of patients had an implant size of more than 305 ml and less than 10% of patients had drains which were removed the next morning. All patients were followed regularly and no case of implant infection or removal was seen and only 2 patients had slight stretched scars. In one patient, the implant was high riding and no case of the capsular contracture was noticed. Changes in sensation were noted in 21.9% patients at 3 month interval which was reduced to 6.3% at 6 months interval. Similarly no case of rippling or other visible deformity was noted.

**CONCLUSION:**

The extra-glandular periareolar approach for the breast augmentation can be a good option with few side-effects even it is associated with a higher level of surgical expertise.

## INTRODUCTION

Breast augmentation is the most frequent procedure performed according to the 2009 Quick Facts report of the American Society of Plastic Surgeons.^1^ Incision placement in these patients is an important element of the overall strategic surgical plan.^2^ The incision must provide sufficient access to the breast tissue to afford accurate dissection of the pocket; to allow easy insertion of the implant, and to provide for precise hemostasis. At the same time, the incision should be placed there and the resulting scar will be inconspicuous and well hidden. 

The periareolar incision is associated with various advantages. The controlled development of pocket under direct vision is the most important advantage. The controlled release of the pectoralis major muscle fibers can be performed as needed. The subglandular and subfascial planes can be developed with equal facility.^3^^,^^4^ The precise control of hemostsis provides a relatively dry and blood-free pocket which in turn results in reduced rate of capsular contracture.^5^^,^^6^ The additional specific advantages that make it the preferred technique for many surgeons. By locating the incision directly at the junction between the pigmented skin of areola and the lighter skin of the breast, a very inconspicuous scar is created that heals in an imperceptible fine-line fashion the majority of the time. The scar is only visible when the entire breast is exposed, which obviates the risk of the scar showing when bathing suits are worn. Moreover, the location of the inframammary fold can be positioned with accuracy. Finally, should revisionary surgery be required, it is usually possible to enetr the breast through the existing periareolar scar to provide exposure for such procedures as capsulotomy, capsulectomy, contour placation, and implant exchange. The periareolar approach is associated with potential disadvantages. The most obvious factor is the areolar diameter. A 3 cm wide areolar incision will accommodate a 4.7 cm static incision along the inferior hemisphere of the areola. The following study was conducted in a private setup in patients undergoing breast augmentation with implant by an extra-glandular route. 

## MATERIALS AND METHODS

The study was conducted in a private setup. All the female patients requesting the implant augmentation mammoplasty were enrolled. All cases underwent gel-filled silicone implant augmentation. Those undergoing cohesive implants, saline-implants or fat grafting to the breast were excluded. Similarly patients undergoing augmentation through inframammary, trans-axillary or trans-umbilical route were also excluded. All operations were primary surgeries. 

**Fig. 1:**
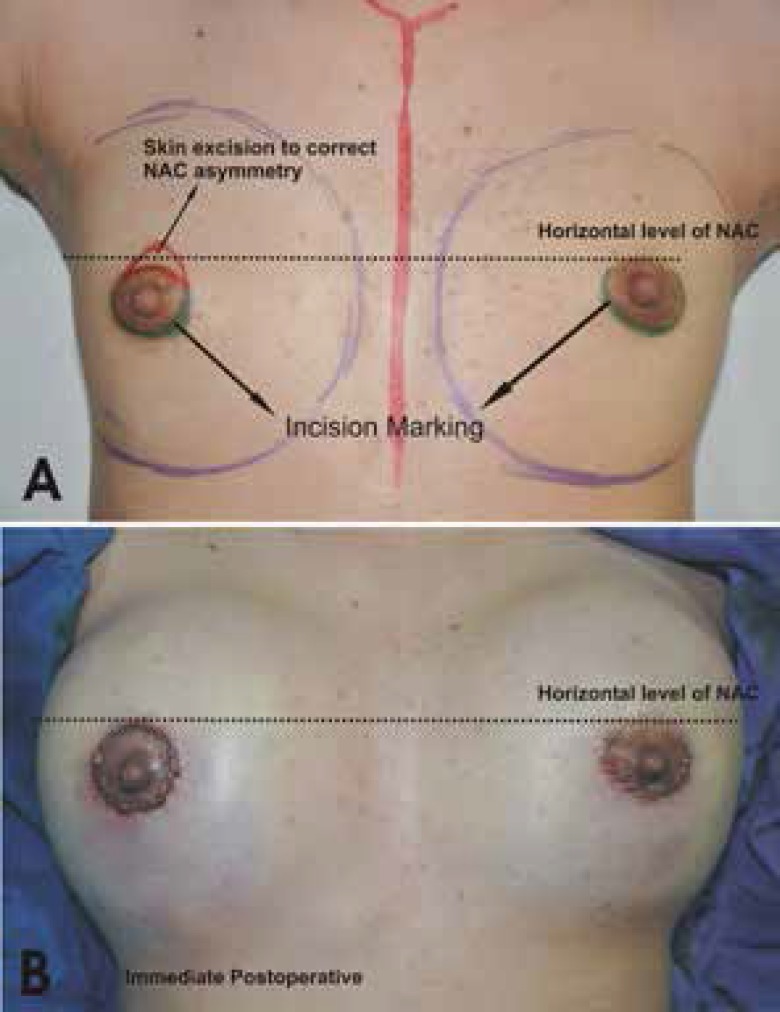
Incision markings

**Fig. 2:**
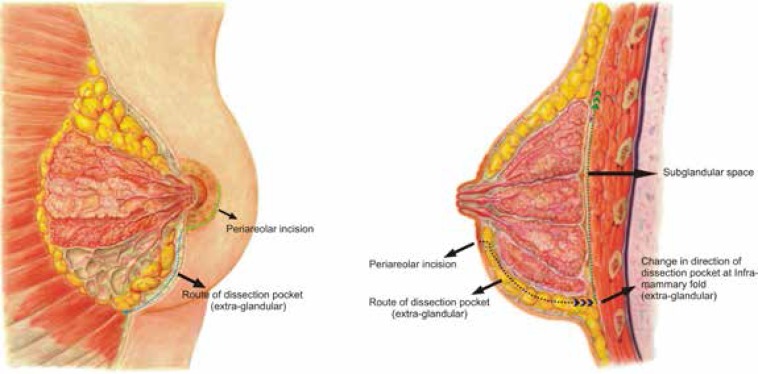
Dissection pattern

**Fig. 3:**
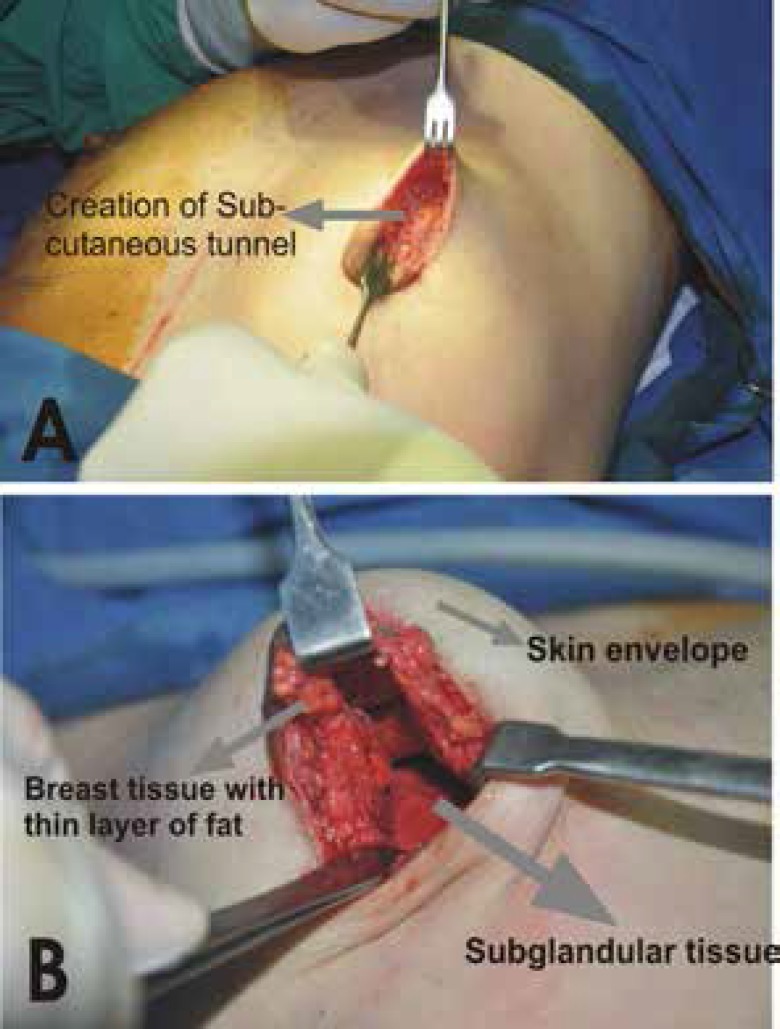
Creation of the pocket

In all patients, the incision was marked preoperatively. The total length varied from 4 to 7 cm. The incision was approximately half to three quarters of the areolar diameter (starting from 3’o clock position to 9’o clock position for half areolar diameter and from 2’ o clock position to 10’o clock position for three quarters incision) (Figure 1). All procedures were performed under general anesthesia. Local infiltration with 1% xylocaine and 1:100,000 epinephrine was performed. After incision of the skin, the subcutaneous tissue plane was dissected. Sharp dissection was performed here towards the inferior pole. Care was taken not to entre the breast tissue and also more importantly not to damage the skin, thereby creating a subcutaneous plane along the breast parenchymal tissue. The dissection was carried till the inframammary fold was reached. Here the dissection was continued in the upwards direction in the subglandular plane to create the pocket (Figure 2). A light-retractor was used to visualize the pocket and the hemostsis was ensured under vision. Once the pocket was created, the sizer was used to assess the pocket size. The implant of the desired size was then introduced in the pocket by milking in finger movements. No instrument was used while inserting the implant in the pocket. Once the implant was in place, three sutures (one in the middle and one on either side) were used to fix the tissue to the inframammary fold (Figure 3). The closure was done in layers with absorbable 3-0 polyglactan sutures. An absorbable 4-0 suture was used for the subcutaneous skin closure, avoiding any stitch removal late on. Sterile strips were then applied. 

The augmented breasts were wrapped in the dressing. Antibiotics were used for the first ten days and oral analgesics were started after 6-8 hours postoperatively. The patients were advised to use sports-bra for 6 weeks. The wired-bra was advised after 8-10 weeks. The patients were followed up routinely and any complication arising was noted and managed accordingly.

## RESULTS

The study was conducted from 2004 to 2010. A total of 32 patients were included in the study. The mean age of the patients was 30.7 years (range=18–52 years). Majority of patients (71.9%) aged 30 years or less. The average incision length was 5.8 cm (ranging from 4 to 7 cm). Fifty percent of patients had an incision length of 6 cm, 21% required 7 cm and 28.1% required 4 and 5 cm. Majority of patients (59.4%) had an implant size of more than 305 ml. Only 9.4% of patients required less than 200 ml size (Table 1). Less than 10% of patients had drains which were removed the next morning within 24 hours. All patients were followed up regularly. Only few complications were noted during the study. No case of implant infection or removal was seen. Only 2 patients had slightly stretched scar. In only one patient, the implant was high riding. No case of capsular contracture was seen during the study. Changes in sensation were noted in 21.9% patients at 3 months interval which was reduced to only 6.3% at 6 months interval. Similarly no case of rippling or other visible deformity was noted. 

**Table 1:** Volumes of implants used (n=32).

**Implant volume**	**No. of patients**	**%**
175	1	3.1
195	2	6.3
215	3	9.4
255	4	12.5
285	3	9.4
305	3	9.4
325	4	12.5
355	5	15.6
385	5	15.6
435	2	6.3

**Fig. 4:**
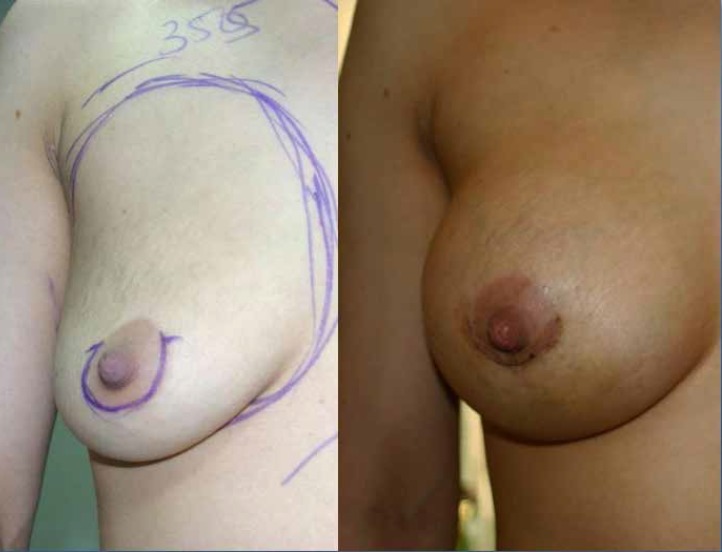
Preoperative and Post operative photographs

**Case 1: **A 30 years old female requested for an increase in size. A 355 ml gel-filled breast implant was placed through peroareolar incision in the sub-fascial plane. The patient was satisfied with the outcome (Figure 4).

**Fig. 5:**
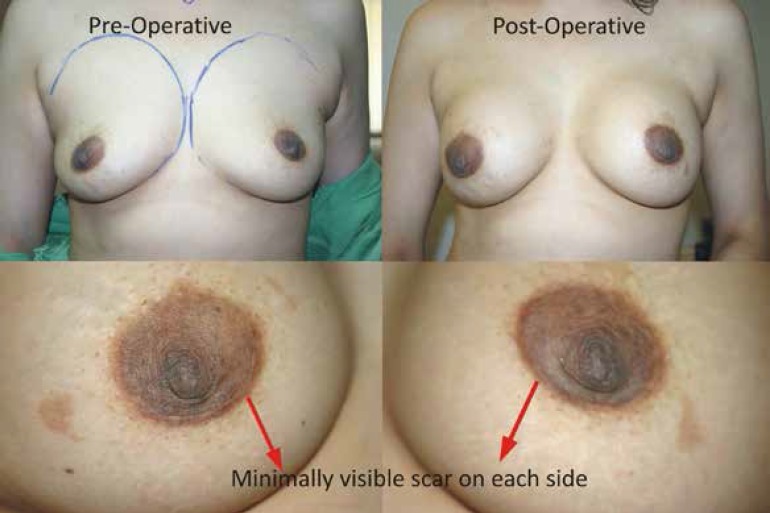
Preoperative and Post operative photographs

**Case 2: **A 31 years old female was interested in augmentation. A 350 ml gel-filled mammary implant was placed via peri-areolar incision in the sub-fascial plane. The patient was satisfied with the post-operative result (Figure 5).

**Fig. 6:**
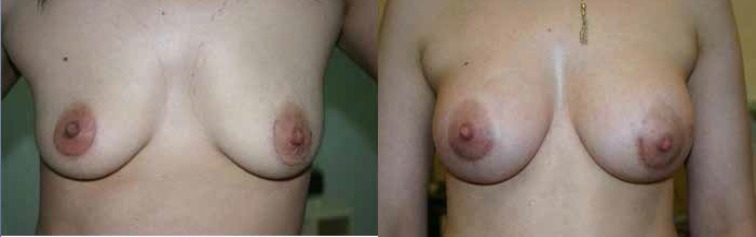
Preoperative and post-operative results of a 39 years old female

**Case 3: **A 39 years old female was interested in increase of the breast size and augmentation in the profile. She underwent augmentation using 385 ml gel-filled implant through peri-areolar incision. The post operative result was satisfactory (Figure 6) 

## DISCUSSION

The periareolar approach for augmentation was first described in 1970’s.^7^^-^^9^ There are two techniques for the pocket dissection, i.e., transparenchymal and periaparenchymal (extra-glandular).^10^ The most straight forward approach to the under side of the breast through a periareolar incision is to dissect directly through the breast to the pectoralis major muscle (Figure 2). At this point, a subglandular, subpectoral or subfascial pocket can be created. This approach is associated, theoretically, with the potential for seeding of the implant surface or the pocket as a result of the direct division of the breast parenchyma. As the ductal system of the breast is exposed, it is possible for bacteria within the ducts to contaminate the implant as it is passed through the incision, leading to biofilm formation within the pocket and hence resulting in a greater potential for the development of capsular contracture.^10^

To avoid these potential dangers of development of capsular contracture, an alternate extra-glandular approach is used to dissect the pocket. As the ductal system and the breast parenchyma is fully avoided from transaction, the chances of contamination are minimal, and hence results in lower chances for the development of capsular contractures. Moreover, the other advantage could be in implant placement, i.e., it is possible to produce the inframammary fold more easily as the suturing is done under vision. The periareolar technique is also associated with another advantage, i.e., any asymmetry of the nipple-areola complex can also be addressed. This technique may be associated with some potential dangers which are related to the degree of difficulty encountered during the pocket dissection and implant placement. It may also result in potential danger of double-bubble appearance, if the lower pole fixation is not done properly.

Another limiting factor for the use of periareolar approach could be the diameter of the areola. However, an areola as small as 25 mm in diameter will allow for the creation of a 4 cm incision along one half of the areolar circumference which is sufficient to allow the passage of the most of the gel-filled implants.^11^ The changes in sensation are only temporary resulting due to nerve stretching from the lateral dissection. A medically placed incision avoids the fourth intercostals nerves, which supplies the sensation to nipple-areolar complex.^12^

Another interesting modification was performed by Miglioro *et al.* using the ‘upside-down’ augmentation in which the authors perform the augmentation through upper periareolar incision and then create the same subfascial tunnel from upside down to the inframammary crease.^13^ Although they present excellent results but the technique may be associated with a few drawbacks. It may be difficult to create the exact plane of the breast tissue at the upper end especially in the presence of the axillary tail of the breast. Moreover, the distance of the dissection to the pocket is more than the distance at the lower pole. The new inframammary crease creation would be more difficult with this ‘upside-down’ technique. The augmentation can be performed in all three planes, i.e., subglandular, subfascial and subpectoral.^14^^-^^16^

The transparenchymal approach was associated with the highest incidence of capsular contractures,^17^ but avoiding the breast tissue contamination can result in the lower incidence of capsular contractures. To reduce these complications in patients with breast ptosis, a separate inframammary incision was used by Wiener giving the patients two scars (11.6%).^7^ This extra-incision could have been avoided if the extra-glandular approach was adopted. So the extra-glandular periareolar approach for the breast augmentation can be a better option with a fewer side-effects but is associated with a higher level of surgical expertise. 

## CONFLICT OF INTEREST

The authors declare no conflict of interest.

## Footenote

Please cite this paper as:

Mohmand MH, Ahmad M. Periareolar Extra-Glandular Breast Augmentation. World J Plast Surg 2013;2(2): 93-98.

## References

[1] The American Society of Plastic Surgeons (2010). 2009 Quick Facts: Cosmetic and Reconstructive Plastic Surgery Trends. The American Society of Plastic Surgeons.

[2] Hammond DC (2009). The periareolar approach to breast augmentation. Clin Plast Surg.

[3] Graf RM, Bernardes A, Rippel R, Araujo LRR, Damasio RCC, Aversvald A (2003). Subfascaial breast implant: a new procedure. Plast Reconstr Surg.

[4] Spear SL, Bulan EJ (2001). The medical periareolar approach to sub-muscular augmentation mammaplasty under local anaesthesia; a 10 year follow up. Plast Reconstr Surg.

[5] Milojevic B (1983). Unilateral fibrous contracture in augmentation mammoplasty. Aesth Plast Surg.

[6] Hipps CJ, Raju R, Straith RE (1978). Influence of some operative and postoperative factors on capsular contracture around breast prostheses. Plast Reconstr Surg.

[7] Owsley Jr JQ, King D (1974). Silicone gel breast augmentation with a periareolar incision. Chir Plastics (Berl).

[8] Jones FR, Tauras AP (1973). Periareolar incision for augmentation mammoplasty. Plast Recosntr Surg.

[9] Jenny H (1974). Areoalr approach to augmentation mammaplasty. Plast Recosntr Surg.

[10] Hammond DC (2009). Atlas of Aesthetic Breast Surgery.

[11] Spear SL, Beckenstein M, Spears SL (1998). The periareolar approach to augmentation mammaplasty. Surgery of the breast: Principles and art.

[12] Walden JL, Aston SJ, Steinbrech DS (2009). Breast Augmentation. Aesthetic Plastic Surgery.

[13] Migliori F (2011). Upside-Down augmentation mastopexy. Aesth Plast Surg.

[14] Handricks H (2007). Complete sub muscular breast augmentation: 650 cases managed using an alternative surgical technique. Aesth Plast Surg.

[15] Khalili MAS, Scholze R, Morgan WR, Metcalf JD (2004). Subfascial periareolar augmentation mammaplasty. Plast Recosntr Surg.

[16] Hunstand JP, Webb LS (2010). Subfascial breast augmentation: a comprehensive experience. Aesth Plast Surg.

[17] Wiener TC (2011). Relationship of incision choice to capsular contracture. Plast Reconstr Surg.

